# The Real-World Impact of Long-Acting Injectable Cabotegravir-Rilpivirine on Weight and Renal Function

**DOI:** 10.7759/cureus.78384

**Published:** 2025-02-02

**Authors:** Lukas McNaboe, Alvaro Ayala, Julia Kostka, Dorothy Wakefield, Lisa Chirch

**Affiliations:** 1 Department of Medicine, University of Connecticut, Farmington, USA; 2 Department of Medicine, Division of Infectious Diseases, University of Connecticut, Farmington, USA; 3 Statistics, Saint Francis Hospital and Medical Center, Hartford, USA

**Keywords:** antiretroviral therapy and human immunodeficiency virus, cabotegravir rilpivirine, medication induced weight gain, non-randomized observational data, renal function

## Abstract

Despite the continued advancement in the efficacy and convenience of antiretroviral therapy (ART), counseling patients about the metabolic side effects of medications remains an integral task for clinicians. This retrospective study examines changes in weight and renal function in patients receiving long-acting injectable cabotegravir-rilpivirine (LAI CAB/RPV) compared to oral regimens in a real-world setting. In this small set of patients, LAI CAB/RPV led to less weight gain compared to oral integrase inhibitor-based regimens. However, there was no significant change in the estimated glomerular filtration rate (eGFR) between patients who were started on LAI CAB/RPV and those who continued to receive oral regimens with or without tenofovir alafenamide (TAF).

## Introduction

HIV-associated morbidity and mortality have decreased substantially in the era of combination antiretroviral therapy (ART), and people living with HIV (PLWH) may expect to have near-normal lifespans. Metabolic disorders such as diabetes and cardiovascular disease are common conditions in PLWH, as in the general population, although HIV-related inflammation and ART-induced weight gain may represent independent risk factors. The degree to which ART can affect weight varies from drug to drug, and a robust understanding of these effects is key when selecting regimens and counseling patients. 

Integrase strand transfer inhibitors (INSTIs) represent a component of first-line ART in PLWH. Although generally well-tolerated, weight gain is a well-described adverse effect of this therapeutic class, which has limited use in some patients. This trend has been most strongly associated with dolutegravir and bictegravir, and, to a variably lesser extent, with elvitegravir and raltegravir [1,2]. Numerous mechanisms have been proposed to explain these phenomena based on in vitro and in vivo models, including decreasing mitochondrial mass [3], suppression of the melanocortin system [4], and combinations of these and other mechanisms [5,6]. Due to the varying magnitude of this effect across the class and likely multifactorial pathophysiology, the extent to which the most recently approved INSTI, long-acting cabotegravir, is associated with weight gain is unknown.

Initial medication safety data of cabotegravir in non-HIV infected individuals demonstrated no significant weight changes after one year of cabotegravir administrations, and no difference in weight change compared to those receiving placebo injections [7]. Furthermore, as the regimen is delivered intramuscularly, the INSTI-induced orexigenic suppression of the melanocortin-stimulating system proposed by Domingo et al. may only occur to a lesser extent, although direct evidence is limited [8]. More recent data from the SOLAR trial demonstrated no significant differences in weight after one year between patients who switched to long-acting cabotegravir-rilpivirine (LAI CAB/RPV) compared to those who remained on the regimen of bictegravir/emtricitabine/tenofovir alafenamide [9,10].

An additional potential metabolic advantage of LAI CAB/RPV is that it does not contain tenofovir. Tenofovir is a nucleoside analog, which is an effective, generally well-tolerated component of many first-line ART regimens. Due to the low oral bioavailability of the active form, it is available as two prodrugs: tenofovir disoproxil fumarate (TDF) and tenofovir alafenamide (TAF). TDF is associated with weight suppression and renal impairment (i.e., higher serum creatinine); as such its use is limited for patients with estimated glomerular filtration rate (eGFR) <50 mL/min/ 1.7m^2^ [11]. TAF is effective at lower concentrations than TDF, and is associated with less renal impairment, but has been associated with weight gain in ART initiation and switch trials [12,13].

Previous ART switch trials demonstrate weight gain when switching to TAF, which may be due to a loss of the weight-suppressive effects of TDF, instead of an inherently obesogenic characteristic of TAF. The SALSA trial provides evidence for this: it reported that switching from a three-drug TAF-containing regimen to dolutegravir/lamivudine did not result in weight loss [14], although this interpretation may be confounded by the potential for dolutegravir to cause weight gain. In a secondary analysis of the ATLAS trial, patients switching from TDF to LAI CAB/RPV demonstrated improvements in renal markers compared to participants continuing with TDF regimens, suggesting that LAI CAB/RPV is not associated with significant renal toxicities and that PLWH switching from TDF regimens may have improvements in renal markers [15]. As TAF-containing regimens are known to cause less renal toxicity than TDF-containing regimens, it is unclear what effect LAI CAB/RPV has on renal function compared to oral TAF-containing regimens.

CAB/RPV is a long-acting injectable INSTI and non-nucleoside reverse transcriptase inhibitor (NNRTI) combination for the treatment of HIV. As its use becomes more prevalent, it is important to understand its effects on weight and renal function to better inform provider and patient decision-making. We hypothesize that patients who switch from regimens containing an oral INSTI and/or TAF to LAI CAB/RPV will experience reduced weight gain and improved renal parameters.

This article was previously presented as a poster at the 2023 Annual ID Week Conference on October 13, 2023.

## Materials and methods

This was a retrospective chart review of patients at a Ryan White-funded, university-based outpatient infectious diseases clinic. During the study period (between January 21, 2021, and July 31, 2022), 22 patients decided to switch from oral ART to the LAI CAB/RPV. The decision to switch was made after an informed consent-related discussion with their infectious diseases physician; LAI ART was offered to all patients who had a consultation during the study period and who did not have a history of INSTI or NNRTI resistance mutations. All 22 of these patients were included in this study. A comparison cohort of 44 additional patients in the clinic who remained on oral INSTI-based ART was randomly selected. As such, a total of 66 adult patients from this clinic were included in the study, and patients were not assigned to different treatment groups but freely chose their method of receiving ART. The inclusion criteria were as follows: patients aged ≥18 years who were treated for HIV infection with an INSTI and followed in this clinic for management of their HIV infection. Patients whose HIV had been treated for less than six months, those taking an INSTI for pre-exposure prophylaxis, and those undergoing weight reduction surgery during this time frame were excluded.

The following parameters were recorded for each study subject: demographic parameters (age, gender, race, ethnicity); presence of select comorbidities [diabetes, hypertension, chronic kidney disease (CKD), microalbuminuria]; select vital signs (weight, BMI, blood pressure); markers of HIV disease status (CD4 count, viral load); current and prior ART regimens; eGFR (CKD-EPI 2021); presence of prescription medications that may impact eGFR (ACE, ARB, SGLT2 inhibitors), and other metabolic parameters (HDL, LDL, total cholesterol). If available in the electronic medical record, these parameters were obtained for each subject at an index time (T0), as well as at the following time points: two, six, and 12 months after the index time (T1, T2, and T3, respectively). Data were obtained from office visits within four weeks of each time point. The T0 in the LAI group corresponded with the visit of their initial dose of LAI CAB/RPV. For the LAI group, parameters were also obtained at the following time points: six and 12 months before initiating LAI CAB/RPV, to assess longer-term within-subject changes.

Statistical analyses were performed using SAS 9.4 software. Descriptive statistics were calculated for all variables. Categorical variables were compared by study group (LAI vs. oral ART) using chi-square analyses and continuous variables were compared using unpaired t-tests. Weight changes relative to the index time were assessed using paired t-tests. Multivariate models assessed the relationship between weight change (between the index visit and the 12-month visit), and demographic variables. Since eight patients were missing at the 12-month weight measurement, we ran a similar model using the six-month weight for these patients and the 12-month weight for all other patients. 

Since weight measurements occurred at different time points for these patients, this model included the number of months between weight measurements as a covariate. For the group who switched to LAI, a paired t-test was also used to assess weight change between one year before and after switching (i.e., a two-year interval period). Additional statistical analyses were conducted to assess the degree to which TAF may influence a patient's renal function (eGFR). We compared study subjects whose ART regimen included TAF either during the study period (oral ART group) or before switching to LAI with subjects without TAF. Weight change was compared among those on TAF vs. no TAF using t-tests. In addition, we compared weight change by study group while controlling for TAF (Y/N) using a multivariate linear regression. A similar model compared eGFR change by study group while controlling for TAF (Y/N) and race.

This study was approved by the UConn Health Institutional Review Board (approval no: 23X-074-1).

## Results

Patient characteristics

During the study period, 22 patients initiated LAI CAB/RPV, and all of them were included in the analysis. A reference cohort of 44 patients who remained on oral ART was randomly selected. The demographic characteristics of these groups are listed in Table 1. The groups were balanced for sex and ethnicity; however, the oral ART group was significantly older than those who switched to LAI CAB/RPV, and there was a significantly higher proportion of people identifying as White in the oral ART group. No differences in systolic blood pressure or lipid panel measurements were observed. As data collection was dependent on an in-office patient visit within four weeks of the time points of interest, we had unequal sample sizes for the study parameters at different times (Table 2). Select vital signs (blood pressure, weight, BMI) were the most commonly available variables, followed by laboratory markers of HIV disease status. Other lab parameters of interest were less commonly available as they are not otherwise a component of routine follow-up care in PLWH. The specific ART regimens are enumerated in the Appendices, including previous oral ART regimens for those patients who switched to LAI.

**Table 1:** Demographic characreristics Also included are baseline blood pressure and lipid panel measurements. T-tests were used to compare continuous variables (age, blood pressure, lipid profile measurements), and categorical values (sex, ethnicity, race) were compared using chi-square analyses ART: antiretroviral therapy; HDL: high-density lipoprotein; LAI CAB: long-acting injectable cabotegravir; LDL: low-density lipoprotein; SBP: systolic blood pressure; SD: standard deviation

Variables	Oral ART	LAI CAB	P-value
	Group 1, (n=44)	Group 2, (n=22)	
Sex, n (%)			0.71
Male	28 (63.6)	15 (68.2)	
Female	16 (36.4)	7 (31.8)	
Age, years, mean (SD)	52.7 (15.3)	43.4 (12.9)	0.02
Race, n (%)			0.03
Black	16 (36.4)	9 (40.9)	
White	21 (47.7)	4 (18.2)	
Other	7 (15.9)	9 (40.9)	
Ethnicity, n (%)			0.71
Hispanic	16 (36.4)	7 (31.8)	
Non-Hispanic	28 (63.6)	15 (68.2)	
Select vital signs, mean (SD)			
SBP, mmHg			
T0	128.3 (20.8)	128.4 (14.5)	0.98
T3	134.4 (19.6)	124.2 (12.8)	0.08
Lipid profile, mean (SD)			
LDL, mg/dL			
T0	107.4 (20.3)	112.8 (37.2)	0.66
T3	120.7 (38.8)	107 (25.5)	0.64
HDL, mg/dL			
T0	48.1 (18.3)	43.3 (9.7)	0.4
T3	48.8 (16.3)	41.0 (14.1)	0.54
Total cholesterol, mg/dL			
T0	185.3 (33.4)	195.7 (59.3)	0.57
T3	197.6 (50.2)	167.5 (30.4)	0.44

**Table 2:** Sample size availablity of each parameter at each time point The values in this table correspond to the number of available measurements for each parameter at the different time points. T0 is the index time when LAI was initiated in the LAI group. For time points at and after T0, the total number of data points obtained (n) are listed as “both groups (LAI, oral)”. For the time points before T0, only data for the LAI group were obtained. The time points T-3 and T-2 correspond to office visits 12 and six months before starting LAI ART, for the group who switched. The time points T2 and T3 correspond to office visits six and 12 months after the index time BMI: body mass index; eGFR: estimated glomerular filtration rate; HDL: high-density lipoprotein; LAI CAB: long-acting injectable cabotegravir; LDL: low-density lipoprotein

Parameter	T-3 (-12 mo)	T-2 (-6 mo)	T0	T2 (+6 mo)	T3 (+12 mo)
Blood pressure, mmHg	22	9	64 (22, 42)	54 (16, 38)	55 (9, 46)
Weight, Kg	22	18	66 (22, 44)	54 (21, 33)	58 (15, 43)
BMI, kg/m^2^	22	18	66 (22, 44)	54 (21, 33)	58 (15, 43)
HIV RNA, copies/mL	14	14	55 (21, 34)	42 (14, 28)	46 (9, 37)
CD4, cells/mL	18	15	54 (21, 33)	37 (13, 24)	43 (7, 36)
LDL, mg/dL	8	5	28 (13, 15)	10 (1, 9)	15 (2, 13)
HDL, mg/dL	8	5	28 (14, 14)	12 (1, 11)	14 (2, 12)
Total cholesterol, mg/dL	8	5	29 (14, 15)	12 (1, 11)	14 (2, 12)
eGFR, mL/min/1.73 m^2^	16	15	53 (22, 31)	40 (14, 26)	43 (11, 32)

Weight gain, markers of HIV status, and eGFR

**Figure 1:**
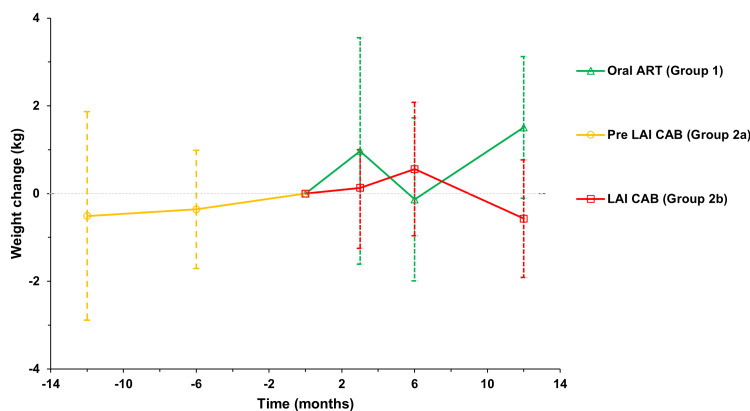
Weight change over time in patients taking oral ART or LAI CAB This figure shows the mean change in weight for each group relative to the index time T0. Note that the sample sizes for the groups at different time points are different as patients may not have had an office visit at which weight was measured near each time point ART: antiretroviral therapy; LAI CAB: long-acting injectable cabotegravir

The between-group analysis for the study parameters of interest is enumerated in Table 3. The groups’ weight and BMI did not significantly differ at any time point, although the LAI group had consistently higher mean weights throughout the study period. Weight change between the index visit and 12-month follow-up showed that the group on oral ART (n=43) gained an average of 1.51 kg, and the group who switched to injectable therapy (n=15) lost an average of 0.57 kg (p=0.045). Within the LAI group, there was no significant weight change over the period one year before and after starting LAI (Figure 1). Regression results showed no relationship between weight change and covariates treatment and TAF usage. At T0, the LAI group had significantly more patients with CD4 ≥400 compared to the oral ART group (p<0.01). In contrast, this difference was not significant at T3. No differences were found for HIV RNA at T0 or T3 (Table 3). The LAI group had higher eGFR than the oral ART group at the time of initiating LAI therapy (p=0.05); this difference was not significant at T3. 

**Table 3:** Outcome variables for groups taking oral ART vs. LAI CAB Continuous variables were compared using T-tests (BMI, weight, renal function). Weight change was compared using paired T-tests. The variables reported to describe HIV disease status were treated as categorical and were compared using chi-square testing. Please refer to Table 2 for a complete description of the sample sizes used for each comparison ART: antiretroviral therapy; BMI: body mass index; eGFR: estimated glomerular filtration rate; LAI CAB: long-acting injectable cabotegravir; SD: standard deviation

Variables	Oral ART (Group 1)	LAI CAB (Group 2)	P-value
				Vital signs			
BMI, kg/m^2^, mean (SD)			
T0	27.4 (4.9)	28.2 (6.4)	0.55
T1	25.7 (5.1)	28.6 (6.2)	0.2
T2	27.0 (4.6)	29.0 (6.5)	0.19
T3	27.8 (5.2)	28.5 (6.7)	0.7
Weight, kg, mean (SD)			
T0	79.6 (16.7)	84.4 (21.7)	0.33
T1	71.6 (16.0)	84.9 (21.2)	0.08
T2	81.1 (16.6)	86.3 (21.5)	0.32
T3	80.8 (16.9)	85.5 (21.7)	0.4
Change: T0 to T3	1.5 (5.2)	-0.6 (2.4)	0.05
Outcome variables			
HIV disease status, n (%)			
CD4 count at T0, cells/mL			<0.01
<400	9 (27.3)	0 (0.0)	
≥400	24 (72.7)	21 (100.0)	
CD4 count at T3, cells/mL			0.31
<400	8 (23.5)	0 (0.0)	
≥400	26 (76.5)	7 (100.0)	
HIV RNA at T0, copies/mL			0.52
<200	32 (94.1)	21 (100.0)	
≥200	2 (5.9)	0 (0.0)	
HIV RNA at T3, copies/mL			1.00
<200	34 (97.1)	9 (100.0)	
≥200	1 (2.9)	0 (0.0)	
Renal function, mean (SD)			
eGFR, mL/min/1.73 m^2^			
T0	77.6 (22.1)	90 (21.2)	0.05
T3	81.7 (26.2)	88.1 (15.8)	0.45

In a comparison of study subjects whose ART regimen included TAF either during the study period (oral ART group) or before switching to LAI with subjects without TAF, there was no significant difference in weight change or eGFR at baseline, six months, and 12 months or change in eGFR between baseline and 12 months. When adjusted by treatment and race, there was no significant relationship between change in eGFR and TAF usage (p=0.66) or treatment (p=0.13). In contrast, Black patients’ eGFR decreased (-4.5) while White patients’ eGFR increased (10.8) (p=0.02). No differences were found between Hispanic patients and White or Black patients. These results were similar to models that did not include TAF as a covariate.

## Discussion

These results provide additional real-world insights into the effects of LAI CAB/RPV on weight change and renal function relative to other ART regimens in the context of a university-based outpatient clinic. Initial medication safety investigations and more recent clinical trial data did not observe changes in the mean weight of treatment and control groups by unpaired t-test comparison, as was observed here [7,9,10]. By comparing the change in weight relative to an index time using pairwise comparisons, our data suggest that LAI CAB/RPV was not associated with weight gain to the same extent as oral ART regimens in this cohort and may even be associated with weight loss. This may be due in part to a more heterogeneous reference cohort with respect to baseline oral regimens. In the SOLAR trial, for example, the comparison cohort received bictegravir/emtricitabine/TAF, while in the current study, 23% of the reference cohort received regimens containing DTG, the most obesogenic INSTI [1]. These findings should be interpreted with caution given the variability in potentially relevant baseline demographic factors between groups, including race and age, as well as the small size of comparator groups and relative lack of statistical power.

In terms of renal function, TAF has improved renal outcomes compared to TDF. While the ATLAS trial suggested that LAI CAB/RPV may be associated with less renal toxicity than TDF-containing regimens, little is known as to whether the same is true of TAF-containing regimens. Our limited data suggest no significant difference in renal function between LAI CAB/RPV and TAF-containing regimens over the study period. Although most relevant baseline characteristics were similar, the LAI group was both younger and had a higher representation of patients identifying as Black; based on population-level data, these characteristics would likely be associated with improved and worsened renal function, respectively. The relative magnitude of each of these possible confounding variables is unclear. Further real-world data assessing longer-term renal outcomes in a larger cohort of patients on LAI is needed, as this represents one clinically important nucleoside-sparing regimen for an at-risk aging population living with HIV. As more PLWHs continue to age with chronic illnesses, often receiving a host of other nephrotoxic medications, reliable data on the relative renal safety of newer regimens will be of critical importance.

An important limitation of this study was the variability in the availability of follow-up data at the different time points, especially at interval visits between the index time and the 12-month follow-up. Our ability to extract specific data at specific time points relative to medication switch was significantly limited by the retrospective nature of the study. It is notable, however, that the unpaired comparisons of weight are consistent with previously reported findings. Understanding the magnitude of this effect relative to other ART regimens necessitates longer-term follow-up of a larger sample size, with reliable data point extraction at specific time points. The latter aspect would be greatly facilitated by a prospective study design. Regarding sample size, the inclusion of patients who receive care at multiple different clinics would enable analysis with a larger scope, as well as more generalizability.

Additional considerations impacting the generalizability of this data include the demographic differences observed between the treatment and control groups. Specifically, the mean age of the LAI group was nearly 10 years younger than those who remained on oral ART. This is not unexpected as older patients may be more likely to take additional daily medications, and therefore would not see an injectable alternative to their ART as more convenient. The degree to which this observed age gap influences the differences in weight change that was observed is unclear. While older adults may be predisposed to weight gain due to declining metabolic rates, there is also muscle atrophy associated with normal aging [16], and, as such, additional investigation including age-matched controls would help elucidate any effect size attributable to LAI CAB/RPV. Regarding race, the prevalence of overweight and obesity is higher in communities of color. Thus, a higher representation of these groups in the LAI CAB group with weight gain would be expected, but this was not found.

## Conclusions

This study demonstrated relatively less weight gain associated with LAI CAB/RPV than with oral INSTI-based regimens in a small cohort, particularly after more than 12 months. These results contrast with recently presented clinical trial data that failed to demonstrate significant differences in weight, BMI, or body composition in patients on LAI CAB/RPV vs. those taking bictegravir/emtricitabine/TAF. There was no significant change in eGFR between patients who were started on LAI and those who continued on oral regimens, with or without TAF. As discussed above, the major limitations of this study are its small sample size, reliance on the availability of data from office visits, and its retrospective design, which prohibit any interpretation of causality and limit the ability to draw firm conclusions. Further data is needed to advance clinical knowledge and practice concerning LAI ART in PLWH.

## Appendices

**Table 4:** Antiretroviral regimens of the patients in this study, including prior regimens of those who switched to LAI CAB Oral lead-in not applicable for non-switch patients

LAI CAB (1 = switched, 0 = remained on oral ART)	Anchor	Backbone 1	Backbone 2	Others	Oral CAB/RPV lead-in
1	DTG	RPV			y
1	BIC	FTC	TAF		y
1	BIC	FTC	TAF		y
1	EVG-c	FTC	TAF		y
1	BIC	FTC	TAF	Maraviroc	y
1	RPV	FTC	TAF		y
1	DRV-c	FTC	TAF		y
1	BIC	FTC	TAF		y
1	DTG	3TC	ABC		y
1	DTG	3TC			n
1	DTG	3TC			n
1	BIC	FTC	TAF		y
1	BIC	FTC	TAF		n
1	EVG-c	FTC	TAF		n
1	BIC	FTC	TAF		y
1	DTG	3TC	ABC		y
1	DTG	FTC	TAF		n
1	RPV	FTC	TAF		n
1	BIC	FTC	TAF		n
1	BIC	FTC	TAF		y
1	DTG	RPV			y
1	BIC	FTC	TAF		n
0	DTG	FTC	TAF		
0	DTG	3TC			
0	BIC	FTC	TAF		
0	DTG	3TC	ABC		
0	DTG	RPV			
0	DTG	RPV			
0	EVG-c	FTC	TAF		
0	BIC	FTC	TAF		
0	BIC	FTC	TAF		
0	EVG-c	FTC	TAF		
0	EVG-c	FTC	TAF		
0	BIC	FTC	TAF		
0	BIC	FTC	TAF		
0	BIC	FTC	TAF		
0	BIC	FTC	TAF		
0	EVG-c	FTC	TAF		
0	BIC	FTC	TAF		
0	EVG-c	FTC	TAF		
0	EVG-c	FTC	TAF		
0	DTG	3TC	ABC		
0	DTG	3TC	ABC		
0	BIC	FTC	TAF		
0	BIC	FTC	TAF		
0	BIC	FTC	TAF		
0	EVG-c	FTC	TAF		
0	EVG-c	FTC	TAF		
0	BIC	FTC	TAF		
0	EVG-c	FTC	TAF	DRV-c	
0	BIC	FTC	TAF		
0	BIC	FTC	TAF		
0	BIC	FTC	TAF		
0	BIC	FTC	TAF		
0	DTG	3TC	ABC		
0	BIC	FTC	TAF		
0	EVG-c	FTC	TAF		
0	EVG-c	FTC	TAF		
0	BIC	FTC	TAF		
0	DTG	3TC	ABC		
0	EVG-c	FTC	TAF		
0	EVG-c	FTC	TAF		
0	BIC	FTC	TAF		
0	BIC	FTC	TAF	DRV-c	
0	DTG	3TC	ABC		
0	BIC	FTC	TAF		

## References

[1] Goldberg RN, Kania AT, Michienzi SM, Patel M, Badowski ME (2021). Weight gain in incarcerated individuals living with HIV after switching to integrase strand inhibitor-based therapy. J Int Assoc Provid AIDS Care.

[2] Grabar S, Potard V, Piroth L (2023). Striking differences in weight gain after cART initiation depending on early or advanced presentation: results from the ANRS CO4 FHDH cohort. J Antimicrob Chemother.

[3] Vakili S, Paneru B, Guerrier CM (2022). Altered adipose tissue macrophage populations in people with HIV on integrase inhibitor-containing antiretroviral therapy. AIDS.

[4] Domingo P, Villarroya F, Giralt M, Domingo JC (2020). Potential role of the melanocortin signaling system interference in the excess weight gain associated to some antiretroviral drugs in people living with HIV. Int J Obes (Lond).

[5] Díez-Domingo J, Martín IO, Sanz AB (2006). Rotavirus gastroenteritis among children under five years of age in Valencia, Spain. Pediatr Infect Dis J.

[6] Wood BR, Huhn GD (2021). Excess weight gain with integrase inhibitors and tenofovir alafenamide: what is the mechanism and does it matter?. Open Forum Infect Dis.

[7] Landovitz RJ, Zangeneh SZ, Chau G (2020). Cabotegravir Is not associated with weight gain in human immunodeficiency virus-uninfected individuals in HPTN 077. Clin Infect Dis.

[8] McMahon C, Trevaskis JL, Carter C (2020). Lack of an association between clinical INSTI-related body weight gain and direct interference with MC4 receptor (MC4R), a key central regulator of body weight. PLoS One.

[9] Tan DH, Antinori A, Eu B (2023). Weight and metabolic changes with long-acting cabotegravir and rilpivirine or bictegravir/emtricitabine/tenofovir alafenamide. J Acquir Immune Defic Syndr.

[10] Ramgopal MN, Castagna A, Cazanave C (2023). Efficacy, safety, and tolerability of switching to long-acting cabotegravir plus rilpivirine versus continuing fixed-dose bictegravir, emtricitabine, and tenofovir alafenamide in virologically suppressed adults with HIV, 12-month results (SOLAR): a randomised, open-label, phase 3b, non-inferiority trial. Lancet HIV.

[11] Wassner C, Bradley N, Lee Y (2020). A review and clinical understanding of tenofovir: tenofovir disoproxil fumarate versus tenofovir alafenamide. J Int Assoc Provid AIDS Care.

[12] Venter WD, Moorhouse M, Sokhela S (2019). Dolutegravir plus two different prodrugs of tenofovir to treat HIV. N Engl J Med.

[13] Sax PE, Erlandson KM, Lake JE (2020). Weight gain following initiation of antiretroviral therapy: risk factors in randomized comparative clinical trials. Clin Infect Dis.

[14] Llibre JM, Brites C, Cheng CY (2023). Efficacy and safety of switching to the 2-drug regimen dolutegravir/lamivudine versus continuing a 3- or 4-drug regimen for maintaining virologic suppression in adults living with human immunodeficiency virus 1 (HIV-1): week 48 results from the phase 3, noninferiority SALSA randomized trial. Clin Infect Dis.

[15] Benn P (2021). Renal/bone outcomes after long-acting cabotegravir-rilpivirine in ATLAS + ATLAS-2M. CROI.

[16] Wilkinson DJ, Piasecki M, Atherton PJ (2018). The age-related loss of skeletal muscle mass and function: measurement and physiology of muscle fibre atrophy and muscle fibre loss in humans. Ageing Res Rev.

